# A review of minimal access surgery provision and training within the United Kingdom

**DOI:** 10.1007/s11701-024-01973-z

**Published:** 2024-05-31

**Authors:** Matthew W. E. Boal, Jessica J. Tan, Shameena Sangarapillai, Vimaladhithan Mahendran, Anuradha Thrikandiyur, Alexander Wilkins, Ata Jaffer, Nayaab Abdul-Kader, Hamzah I. Choudhry, Rikesh Patel, Andrew R. Day, Nader K. Francis, Tamsin E. M. Morrison, the Association of Laparoscopic Surgeons of Great Britain and Ireland (ALSGBI) Academy Research Group, London, UK

**Affiliations:** 1 Association of Laparoscopic Surgeons of Great Britain and Ireland (ALSGBI) Academy, London, UK; 2 The Griffin Institute, Northwick Park and St Marks Hospital, Harrow, UK; 3 University College London, London, UK; 4 Surrey and Sussex Healthcare NHS Foundation Trust, Redhill, UK; 5 Gloucestershire Hospitals NHS Foundation Trust, Gloucester, UK; 6 Hull University Teaching Hospitals NHS Trust, Hull, UK; 7 Stockport NHS Foundation Trust, Stockport, UK; 8 Yeovil District Hospital, Somerset NHS Foundation Trust, Yeovil, UK

**Keywords:** Robotic surgery, Minimal access surgery, Training, Health care surveys

## Abstract

When combined with healthcare pressures, the exponential growth of robotic-assisted surgery (RAS) has impacted UK-based training outcomes, including the learning curve to competency. Aim: To ascertain the current provision of RAS and investigate differences in access to minimal access surgical (MAS) facilities and training across the UK. A two-armed electronic survey was conducted. The first arm questioned clinical leads regarding robotic practice and future training provisions. The second investigated trainee and trainers’ perceptions of MAS training and facilities. 64% (52/81) of responding trusts utilise a robotic system. The majority (68% [55/81]) have plans to expand or acquire a system within 3 years. 171 responses from 112 UK and Republic of Ireland hospitals were collected for Arm 2. Laparoscopic categories queried whether trainees had access to a formal curriculum, training days and sim-boxes. Most consultants (51.9%) and trainees (51.6%) reported that there was no formal local training curriculum for robotic surgery. Combined responses demonstrated 42.1% (*n* = 195/463) said “yes”, 39.5% (*n* = 183) “no” and 18.4% (*n* = 85) “don’t know”. For combined robotic categories (simulation, training days and operative lists) 28.3% (*n* = 134/473) responded “yes”, 51.6% (*n* = 244) said “no” and 20.1% (*n* = 95) said “don’t know”. This study provides insight into the current provision of robotic-assisted surgery at UK trusts and highlights the need to facilitate regular clinical training and equitable access to MAS simulation within a formal curriculum. This may aid regulation of training in parallel with the expansion of robotic practice and avoid a significant skill acquisition gap and risks to patient safety.

**Supplementary Information:**

The online version contains supplementary material available at 10.1007/s11701-024-01973-z.

## Introduction

General Surgical trainees within the United Kingdom (UK) embark on a minimum 8-year programme to gain the Certificate of Completion of Training (CCT), which requires specialty-specific operative proficiency. Trainees’ operative experience has decreased in recent years due to a multitude of factors including the COVID-19 pandemic [1], waiting list initiatives and increasing pressures within the healthcare system [2] that preclude operative training opportunities. In addition, with the development of more complex endoscopic interventions and the rise of robotic-assisted surgery (RAS), surgical trainees are expected to acquire new skills and learn more challenging techniques within the same time frame.

Fu et al. recently demonstrated that the median number of days per week that UK surgeons perform elective surgery is 50% less than their global counterparts [3]. With reduced operating frequency, the need for UK-based surgeons to undergo efficient, competency-based surgical training is comparatively higher [3]. Understandably, much like the introduction of laparoscopic surgery, the current priority within robotic General Surgery is to ensure that Consultants are trained to competency. However, with an increasing number of procedures being performed robotically by surgeons who may be relatively new to this modality, acquiring sufficient Minimal Access Surgical (MAS) training for the current and upcoming generation has become more challenging.

Surgical simulation can reduce learning curves [4–7] and represents an opportunity for surgeons in training to reach competencies within the defined time frame. However, many are limited by a study budget, despite requirements to fund expensive and desirable courses.

Considering this, and the knowledge that existing pressures on the UK healthcare system are likely to take a significant time to improve, efforts from the surgical community are required  to target a reduction in learning curves and take the initiative on the provision of high-quality training.

Before addressing these needs, it must first be ascertained what the training landscape is like within the United Kingdom, by identifying gaps and potential inequalities in the provision of MAS training.

## Aims

There were two aims of this study; the first was to ascertain the current level of robotic-assisted surgery provision and training opportunities from clinical leads at UK hospital trusts. Secondly, we aimed to capture the perceptions of surgeons in training on the provision of minimal access training opportunities for both laparoscopic and robotic surgery, to identify gaps and offer possible curriculum-directed solutions.

## Methods

A survey was designed with two arms and conducted using electronic survey platforms (Supplementary Fig. 1 and 2). A second round of dissemination was performed for both surveys to increase the total number of responses. Data collection was completed in August 2023.

The first arm invited clinical leads from every trust in Great Britain and Ireland to respond, surveying the current and future provision (3-year plan) of robotic-assisted surgery and the availability of training on robotic systems. Trusts were not surveyed regarding simulator access or purchases. The second phase of the survey was sent via email to multiple surgical societies including the annual conference of the Association of Laparoscopic Surgeons of Great Britain and Ireland (ALSGBI). Survey categories were split into laparoscopic and robotic. Within the laparoscopic section, respondents were asked whether training was guided by a formal curriculum, and whether they had regular access to training days and simulation boxes. The robotic categories queried whether trainees had regular access to simulation, training days and operating lists. Response options were positive (“yes”) or negative (“no” and “don’t know”). The data analysis was purely descriptive, and percentages were provided. When reviewing grade, all specialty answers were combined along with each category above, therefore, representing a higher response number than the total survey participants. Some questions were not applicable to all respondents, especially when pertaining to grade-based experience.

## Results

### UK trusts robotic provision responses

**Fig. 1:**
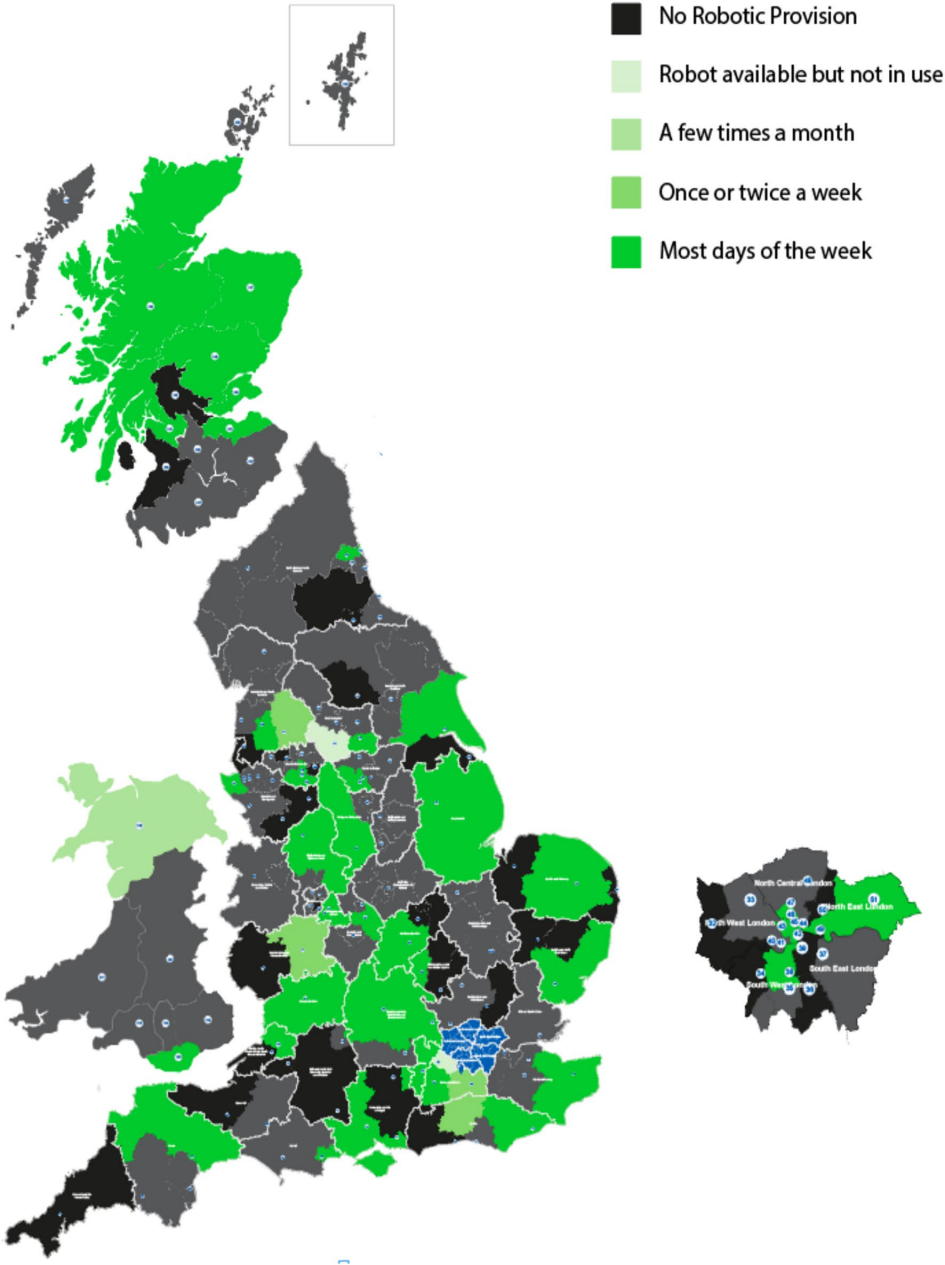
Frequency of use of robotic-assisted surgery. Dark grey areas represent non-respondents

150 trusts were approached with an overall response rate of 54% (81/150), Scotland had the highest (57.1% [8/14]) and Wales the lowest (28.6% [2/7]). Sixty-four percent (52/81) of responding trusts had a robotic system available. Of these, 75% (39/52) were exclusively using an Intuitive Surgical® system and5 had Versius^®^, by Cambridge Medical Robotics (CMR) Surgical. Six trusts had both Intuitive® and the CMR robotic platforms, with one trust in London additionally trialling Medtronic’s Hugo^™^ RAS system.Two trusts did not specify which robotic system they had in place and two trusts reported that they have a robotic system but that it is not in use.The majority (45/52) reported use of the robot most days of the week (Fig. 1).

Of 92.3% (48/52) of trusts with a robot, at least two specialties utilise the platform. Urology used the robot the most in 93.3% (48/52) of trusts with a platform, followed by Colorectal surgeons (78.8% [41/52]) and Gynaecology (65.4% [34/52]).

**Fig. 2:**
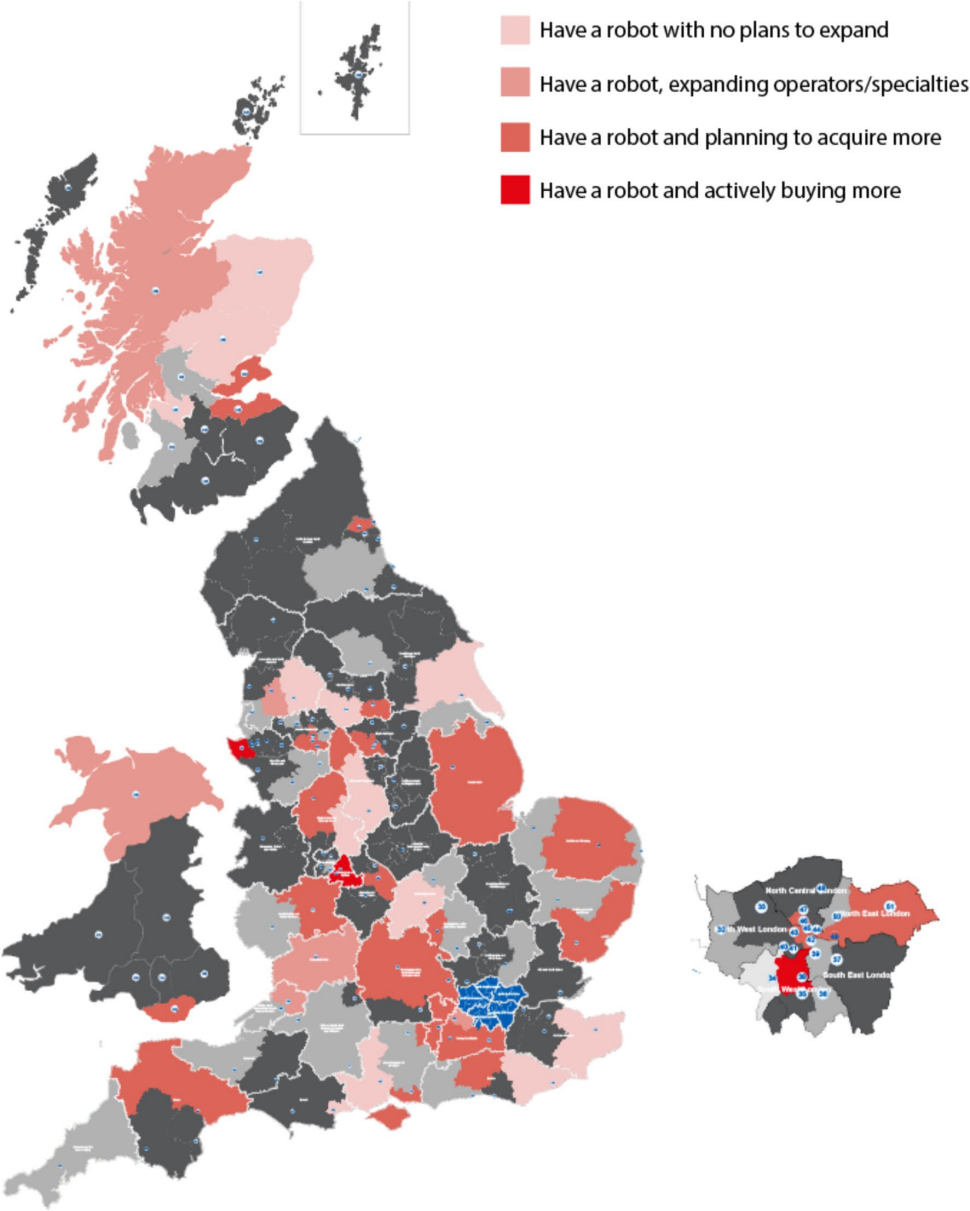
Three-year plan for robotic-assisted surgery in trusts who already have a system. Dark grey areas represent non-respondents, light grey areas represent trusts without a robot currently

Of the 52 responding trusts who had a robot (Fig. 2), only three were actively buying more systems. Most (61.5% [32/52]) were planning to either expand the number of operating surgeons and specialties using the robot within their trust or had plans to acquire more robotic consoles. Most free text responses for 3-year plans included reference to ‘planned expansion’ without further details or stated they were awaiting approval of business plans.

**Fig. 3:**
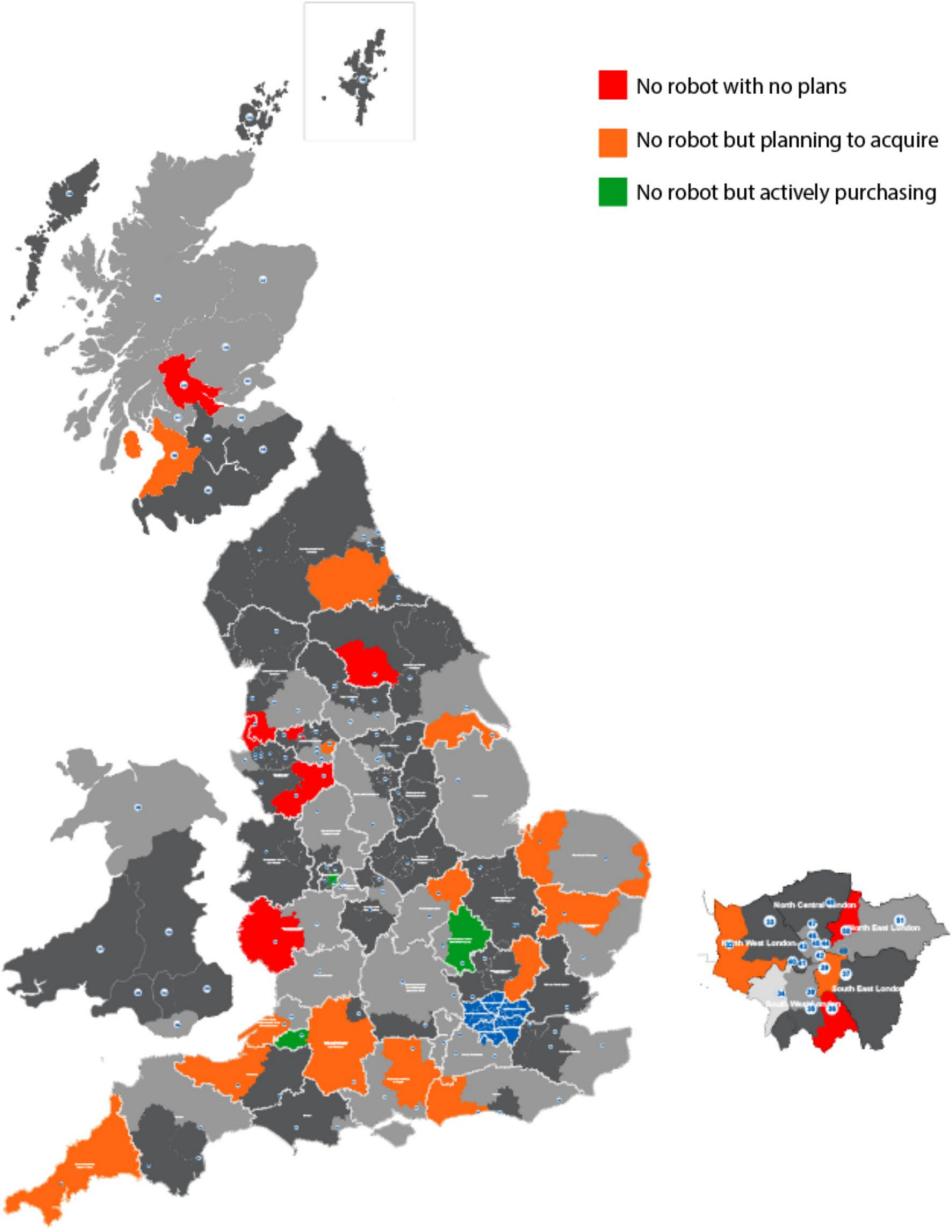
Three-year plan for robotic-assisted surgery in trusts currently without a robot. Dark grey areas represent non-respondents, light grey areas represent trusts with a robotic system

Of the remaining 29 trusts who do not have established RAS services (Fig. 3), only three were purchasing or had already purchased a robot and were awaiting installation. The majority (55.2% [16/29]) are planning to acquire a robot but like the previous respondents, have no “concrete” plans yet. A minority (34.5% [10/29]) had no plans to acquire a robot and one respondent described robotic-assisted surgery as ‘the latest fad’.

**Fig. 4:**
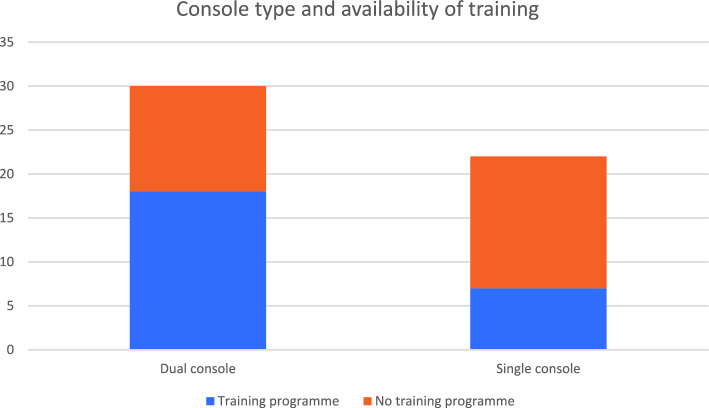
Availability of training programme according to console type

57.7% (30/52) of trusts had a dual console system (Fig. 4). Less than half (48% [25/52]) stated that they had a prescribed robotic training curriculum for trainees. A higher proportion of trusts with dual consoles reported  having a prescribed training curriculum  (60% [18/30])scompared to those with a single console (31.8% [7/22]).

### Minimal access surgery training responses

There were 171 responses from 112 hospitals across the UK and the Republic of Ireland (RoI), with every deanery represented (Supplementary Fig. 1). Scotland represented the highest proportion of respondents (17.5% [30/171]) and the RoI the lowest with one respondent. Ninety respondents (52.6%) were Specialty Registrars, 21.6% (*n* = 37) Core Trainees, 8.2% (*n* = 14) Senior House Officers, 6.4% (*n* = 11) Consultants and 1.8% (*n* = 3) Senior Clinical Fellows. The remaining 9.4% (*n* = 16) were medical students, Foundation doctors, or “other". 84.2% (*n* = 144) represented the specialty of General Surgery, 5.2% (*n* = 9) Urology, 2.4% (*n* = 4) Gynaecology, 2.4% (*n* = 4) Paediatric Surgery, 1.2% (*n* = 2) Ear, Nose and Throat and 0.6% (*n* = 1) Cardiothoracic. The remaining 4% (*n* = 7) were classified as “other”. Ninety-six (56.1%) respondents from 48 hospitals had a robot in their department, of those hospitals 89.6% (*n* = 43) used the Intuitive systems and 10.4% (*n* = 5) Versius® by CMR.

For laparoscopic responses (Supplementary Tables 1 to 3) on trainees’ perspective of whether they have access to a formal training curriculum, responses were split with 29.9% (*n* = 43) saying “yes”, 36.8% (*n* = 53) “no” and 33.3% (*n* = 48) “don’t know”. Sixty-one (42.4%) respondents reported that they had access to laparoscopic simulation days, 50.7% (*n* = 73) said “no” and 6.9% (*n* = 10) responded “don’t know”. Eighty-three (59.7%) respondents stated that they had access to laparoscopic training boxes, 25.9% (*n* = 36) stated “no" and 14.4% (*n* = 20) replied with “don’t know”.

When combining survey domains and responses for all trainee grades and consultant surgeons across specialties regarding regular access to laparoscopic training (curriculum, training days and laparoscopic training boxes), 57.8% (268/463) stated “no” or “don’t know” (Table 1).

**Table 1:** Laparoscopic training access by grades

Grade	Trainee access to laparoscopic training curriculum			Trainee access to laparoscopic simulation days			Trainee access to laparoscopic training boxes		
	Yes	No	Don’t know	Yes	No	Don’t know	Yes	No	Don’t know
SHO (trust grade/JCF)	4	4	6	2	7	5	4	4	6
Core trainee	11	11	15	17	18	1	13	18	3
Specialist trainee	34	27	28	39	45	3	47	32	8
Senior clinical fellow	0	3	2	1	3	1	2	2	2
Consultant	5	5	1	7	2	2	9	2	2

Supplementary Figs. 4 to 6 show the variation across deaneries in access to laparoscopic training across categories.

For robotic responses (Supplementary Tables 4 to 6), 22.5% (*n* = 18) stated that they had access to simulation, while 58.5% (*n* = 47) responded with “no access” and 18.7% (*n* = 15) said “don’t know”. Regarding access to robotic training days, 14.5% (*n* = 21) said “yes”, 73.8% (*n* = 107) responded with “no” and 11.7% (*n* = 17) “don’t know”. 37.6% (*n* = 53) reported access to robotic operating lists, while 31.2% (*n* = 44) reported none and 31.2% (*n* = 44) responded with “don’t know”.

When all trainee grades and Consultant surgeons across specialties were asked about access to robotic training categories (Table 2), 70.6% (307/435) responses were “no” or “don’t know”.

**Table 2:** Robotic training access by grade

Grade	Trainee access to robotic simulation			Trainee access to robotic training days			Trainee access to robotic lists		
	Yes (%)	No (%)	Don’t know (%)	Yes (%)	No (%)	Don’t know (%)	Yes (%)	No (%)	Don’t know (%)
SHO (Trust Grade/JCF)	3	5	4	2	6	6	5	3	6
Core trainee	7	13	6	8	25	4	11	13	13
Specialist trainee	19	42	7	12	69	5	44	29	23
Senior clinical fellow	1	1	1	1	3	1	2	0	2
Consultant	4	5	2	3	5	3	6	5	0

## Discussion

This national survey reflects the current level of provision of robotic-assisted surgery at the trust level across the UK. There appears to be a perceived lack of access in General Surgery to formal, standardised minimal access surgical training that can be used in line with current UK training requirements, particularly within robotics that can complement core competencies.

Robotic surgery is already widespread, even when accounting for the 54% response rate, most trusts now have a robotic system, and at least 27 of the non-responders are known to have a system from hospital websites information. With forecasts of expansion, the trajectory reflected in this data suggests that 80% of trusts may have a robotic system in use by 2027. This is exciting for patient care and surgeon longevity but poses a significant training challenge for this generation of trainees.

Taking a broader view of laparoscopic training, this survey suggests most trainees appear to lack regular access to training days and are not guided by a formal curriculum. The exception to this was access to laparoscopic training boxes, a useful tool for basic skills practice, which was the only category with a majority “Yes” answer. However, 40.3% still do not have access to loaned or given box trainers and this is variable across regions. Ideally, laparoscopic trainer boxes should be guided by a curriculum, but when accessible, most are often used solely through self-directed learning. It appears, therefore, that the landscape has not changed much from ASiT’s 2012 survey and their 2011 report on recommendations for training [8].

Despite laparoscopic surgery being established since the 1990s this survey suggests certain aspects of training appear to be lacking nationally, echoing “The Shape of Training Review” in 2013. Highlighted perceived issues including trainee dissatisfaction with a limited emphasis on training and insufficient time for trainers to provide it. In 2015, this led Health Education England to commission The Royal College of Surgeons to address the issue with the subsequent formulation of the 2015 Improving Surgical Training (IST) report and pilot [9]. The IST pilot sought to improve training and follow the report’s recommendations to embed simulation into training, improve supervision, assessments, and overall trainee experience [10]. However, in 2019 The Association of Surgeons in Training (ASiT) survey found that 55% of trainees felt simulation training was delivered inadequately, 86.2% wanted better access and 98.9% felt it was essential for their training [11]. The survey also highlighted a statement from the head of the Joint Committee of Surgical Training (JCST) in 2013, that simulation is highly desirable although monitoring is necessary first to ensure equitable provision across the country [12].

Efforts to improve training are evident but currently seem not to have significantly impacted the majority of this survey’s respondents. The Royal College of Surgeons England's 2023 census report [13] has again highlighted significant healthcare pressures including surgeon burnout, staff retention, and most trainees surveyed stated that access to theatres is a challenge and there is inadequate time to train. The RCS England has formulated recommendations which are welcomed, with a need for a proactive strategy to address and expedite trainees’ acquisition of minimal access skills to avoid an acquisition lag, narrow the trainee-trainer gap and provide an equal distribution of training.

When comparing responses of the perceived accessibility of robotic and laparoscopic training, there seemed to be a greater deficit within robotic-assisted surgery. This is unsurprising and is likely explained in part by the current capacity and scalability issues. With many Consultant surgeons still on their robotic learning curve, whilst simultaneously providing a clinical service, it is inevitable that training is somewhat precluded. This may have a substantial impact on trainees achieving their specialty-specific operative numbers and procedure-based assessments to reflect safe operative independence. A reduction in trainee suitable caseload will affect their ability to meet Certification of Completion of Training (CCT) requirements within the defined period. This issue requires careful attention from Training Programme Directors, Heads of School and the Royal Colleges to find solutions to mitigate against a suboptimally trained surgical workforce, which would ultimately impact on patient safety.

It is important to note that while formal curricula exist for surgical trainees, which encompass open and MAS techniques, the JCST General Surgery “Index Procedure” list [14] does not specify robotic or laparoscopic procedures despite requiring distinctly different technical skill sets. This can make the standardisation of specific skill requirements and proficiency-based accreditation more complex. It is possible that the inclusion of minimal access surgical skills into formal curricula may help accelerate a more widespread and protected provision of focussed laparoscopic and robotic training.

In the United States, it is mandatory to pass the Fundamentals of Laparoscopic Surgery^®^ (FLS) curriculum [15], a competency-based certification of basic laparoscopic skills. The Fundamentals of Robotic Surgery (FRS) has been developed, evaluated and will likely become mandatory [16–18]. Similarly in the UK, the ALSGBI provides the LapPass^®^ certification training and assessment [19], while the Royal College of Surgeons provides Core, Intermediate and Advanced Laparoscopic Skills courses. Although LapPass^®^ and Core Laparoscopic Skills courses have shared elements, neither are mandatory requirements for entry to or progression in Core or Specialist Surgical Training programmes. At present, robotic courses are being developed and evaluated, but they are certainly not widespread and their costs may not be covered by  a study budget. A BMJ study from 2017 demonstrated that the average trainee spends a minimum of £20,000 to £26,000 [20] by the time of accreditation andthese costs are may be higher with the addition of robotic training. Robotic companies provide device training, with some covering all aspects of a modular curriculum, including proctored live operating [21]. However, these programmes can lack objective assessment,benchmarked accreditationwith potentialeffects on patient safety. There has been consensus that international surgical bodies should be leading training and accreditation in collaboration with industry, as well as an agreement that curricula need to be standardised, fully evaluated and with proficiency-based progression at their core [22].

Health Education England reformed the UK study leave policy in 2018 in the form of a “no cap” policy, understandably with an emphasis that the budget is finite and that some activities that are not required to achieve curriculum outcomes may not be approved (“discretionary study leave”) [23]. Other regions in fact still seem to have lesser amounts allocated to trainees, for example, the study budget in Northern Ireland is £1250 per year [24], which may cover the cost of only one of many mandatory training activities, whilst in Wales it is even lower [25]. There was no easily accessible data from NHS Scotland but anecdotal evidence from trainees suggests it is around £600. Even optimistically,these budgets would only cover half of the average training costs [20]. Despite this, courses such as those listed are considered desirable for job applications and key for the development of MAS technical skills competency.

To anticipate ongoing training issues, virtual reality (VR) and dry model simulation must be considered a key part of any business case upon purchasing new robotic systems, which was not evaluated in this survey. Central simulation hubs are recommended for high-fidelity, expert-led, training days with proficiency-based assessment, however, simple systems-based knowledge and VR simulation competencies can be achieved locally. While this may allow rapid training nationwide with safe progression to mentored console operating, for successful uptake, protected time allocation, with in- and out-of-hours access, is required for robotic novices to acquire these skills. Although there are many pre-clinical, including virtual reality simulation, curricula [16, 26, 27] they are often not standardised or fully evaluated, lacking objective assessment and benchmarking to allow for safer progression to console operating. This represents an additional hurdle for implementation within current surgical training curricula.

Finally, there is a need for an update of existing training curricula to include MAS-specific metrics. Improved MAS training access will help reduce learning curves and enable future surgeons to provide versatile, individualised patient care as well as train the next generation of surgeons whose clinical practice will have increasingly larger proportions of minimal access surgery. The ALSGBI Academy group proposes a formal discussion between key stakeholders in surgical training, including trainee representatives at meetings addressing the  2023 RCS England census report’s fifth recommendation [13]. This could aid the development and implementation of improved minimal-access surgical training across the UK and bring existing curricula in line with current clinical practice.

### Limitations

Despite the high response number of both arms in this survey, there was wide variability in responses when comparing trusts, deaneries and specialties, making comparison difficult and may compromise external validity. Although there is also a risk of response bias, this survey has provided a snapshot view of the current provision of robotic surgery and MAS training, which is dynamic and will most likely rapidly change. This study highlights the already and anticipated widespread use of robotic-assisted surgery in the UK and key areas for improvement in the delivery of minimal access surgical training (Table 3). The expansion of robotic-assisted surgery and minimal access training is dynamic and complex, requiring regular updates reporting on MAS provision for patients and trainee surgeons to ensure fair, equitable healthcare and training delivery.

**Table 3:** Highlighted training issues and recommendations

Issues	Recommendations
1. Perceived lack of access to a formal training curriculum for basic laparoscopic skills across the UK	Consider making LapPass® courses mandatory for Core Trainees applying for General Surgery, deanery representatives ensuring each region can provide faculty, training centres. This may be limited by access to sufficient budget
2. Lack of equal access to laparoscopic simulation training across the UK	Centralised simulation training within each deanery, with consistent, authorised access and formal training days to be part of the teaching curriculum
3. Lack of a formal training curriculum for robotic surgery, across the UK	ALSGBI have developed a 4-day basic robotic skills curriculum An ALSGBI expert panel should discuss scalability, access and expansion of courses at other centres. Deaneries should consider providing appropriate funding to ensure trainees receive adequate introductory training in robotic surgery
4. Lack of access to robotic simulation	If financially feasible, a simulator should be bought and made accessible for all trainees at every trust with a robot. Those who are about to embark on robotic training should be given priority, following completion of a standardised, validated, virtual reality curriculum
5. Lack of access to robotic training lists	Expert consensus is required on robotic training curricula within the UK. Discussion around how to incorporate regular trainee access and graded operative exposure into busy waiting lists. Assisting in robotic procedures should be incorporated into the JCST trainee procedural checklist

## Conclusion

This study provides insight to the current provision of robotic-assisted surgery in the UK and highlights the ongoing need to facilitate formal clinical training and regular, equitable access to laparoscopic and robotic simulation-based training for trainees. Incorporation of a formal laparoscopic and robotic component to the current curricula requirements for surgical trainees is likely to aid the expansion and regulation of MAS training in parallel with technological advances. This is essential to address potential risk to patient safety from significant skill acquisition gaps that may arise between surgical trainees and trainers in the UK.

## Supplementary Information

Below is the link to the electronic supplementary material.

Supplementary Figure1 (PDF 38 KB)

Supplementary Figure2 (DOCX 65 KB)

## Data Availability

The data used to support the conclusions drawn from this study are available on request but are not publicly available in a format that might compromise the anonymity of the survey participants.
