# Supplementary material for: A review of minimal access surgery provision and training within the United Kingdom
Source: J Robot Surg. 2024 May 31;18(1):234. doi: 10.1007/s11701-024-01973-z (PMC11142963; doi:10.1007/s11701-024-01973-z)
Supplement: Supplementary file 1 — Supplementary Figure1 (PDF 38 KB) [file 11701_2024_1973_MOESM1_ESM.pdf]

ALS National Robotic and Training Survey 2022

1. Please tell us the name of your trust or hospital

2. Please tell us a little about your trust. How many beds does your trust have?

- ◯ <100
- ◯ Between 100-250
- ◯ Between 250-500
- ◯ Between 500-750
- ◯ Between 750-1000
- ◯ >1000

3. Over how many sites is your trust spread?

- ☐ 1
- ☐ 2
- ☐ 3
- ☐ 4
- ☐ 5
- ☐ Other (please specify)

4. Does your trust have a robotic system?

- ◯ Yes
- ◯ No

5. If so, is the robotic system currently in use?

- ◯ Yes
- ◯ No
- ◯ Not applicable

6. If the robotic system is in use, on average how often?

- ◯ Most days of the week
- ◯ Once or twice a week
- ◯ A few times a month
- ◯ Once or twice a month
- ◯ Once every few months
- ◯ Currently not in use
- ◯ Not applicable

7. Which specialities are regularly using the robotic system?

- ☐ Colorectal
- ☐ Upper GI
- ☐ General Surgery (ie complex abdominal hernias)
- ☐ Vascular
- ☐ Gynaecology
- ☐ Urology
- ☐ ENT/Maxillofacial
- ☐ Not applicable
- ☐ Other (please specify)

8. Which System(s) do you currently have in place?

	Type
Intuitive System	
CMR Surgical System	
BOWA Medical System	
Distalmotion System	
Medtronic System	

Other (please specify)

9. Do you have a dual robotic console system to aid with training?

- ◯ Yes
- ◯ No

10. Do you have a service contract with the relevant company?

- ◯ Yes
- ◯ No
- ◯ None of the above

11. What is your 3 year plan for robotics, ie: do you plan to expand the type and/or the number of systems you have?

12. Do you have a robotic training program for surgical trainees?

- ◯ Yes
- ◯ No

13. Do your surgical trainees have access to Laparoscopic simulator training in your trust?

- ◯ Yes
- ◯ No

14. If yes, what variety of equipment do you have?

- ☐ Laparoscopic box trainers, ie Inovus
- ☐ Laparoscopic virtual reality trainers, ie Simendo
- ☐ Other (please specify)
- ☐ None of the above

15. If you do have laparoscopic simulator training facilities are they placed in a dedicated area for this purpose?

- ◯ Yes
- ◯ No
- ◯ Not applicable

16. Do your trainees have a prescribed laparoscopic training programme within your trust?

- ◯ Yes
- ◯ No

17. Are you aware of LapPass?

- ◯ Yes
- ◯ No

18. Please provide any other comments you feel relevant:
