# Supplementary material for: A review of minimal access surgery provision and training within the United Kingdom
Source: J Robot Surg. 2024 May 31;18(1):234. doi: 10.1007/s11701-024-01973-z (PMC11142963; doi:10.1007/s11701-024-01973-z)
Supplement: Supplementary file 2 — Supplementary Figure2 (DOCX 65 KB) [file 11701_2024_1973_MOESM2_ESM.docx]

**Journal of Robotic Surgery**

**A review of minimal access surgery provision and training within the United Kingdom**

**Authors:** Matthew W.E. Boal (PhD Research Fellow, matthew.boal@nhs.net)*^1,2,3^, Jessica Tan (General Surgery Registrar, jessica.tan@nhs.net)*^1^, Shameena Sangarapillai (Senior House Officer, shameena.sangarapillai1@nhs.net)^1^, Vimaladhithan Mahendran (General Surgery Registrar, vimaladhithan.m@gmail.com)^1,5^, Anu Thrikandiyur (General Surgery Registrar, anu1593@gmail.com)^1^, Alexander Wilkins (General Surgery Registrar, alexander.wilkins@nhs.net)^1,6^, Ata Jaffer (Consultant Urological Surgeon, atajaffer@nhs.net) ^1,7^, Nayaab Abdul-Kader (General Surgery Registrar, n.abdulkader@nhs.net)^1^, Hamzah Choudhry (Foundation Doctor, hamzah_choudhry@hotmail.com)^1^, Rikesh Patel (Consultant Colorectal Surgeon, rikeshpatel@doctors.net.uk)^1^, Andrew Day (Consultant Colorectal Surgeon, andrew.day1@nhs.net) ^1,4^, Nader K Francis (Consultant Colorectal Surgeon and Director of Training, n.francis@griffininstitute.org.uk)^1,2,8^, Tamsin E.M. Morrison (General Surgery Registrar, ALSGBI Academy President, tamsin.morrison@doctors.org.uk)^1^

On behalf of the Association of Laparoscopic Surgeons of Great Britain and Ireland (ALSGBI) Academy Research Group

*Joint co-authors

**Affiliations:**

1. Association of Laparoscopic Surgeons of Great Britain and Ireland (ALSGBI) Academy
2. The Griffin Institute, Northwick Park and St Marks Hospital, UK
3. University College London, UK
4. Surrey and Sussex Healthcare NHS Foundation Trust, UK
5. Gloucestershire Hospitals NHS Foundation Trust, UK
6. Hull University Teaching Hospitals NHS Trust, UK
7. Stockport NHS Foundation Trust, UK
8. Yeovil District Hospital, Somerset NHS Foundation Trust, UK

**Corresponding author**: Miss Tamsin Morrison, ALSGBI Academy President, [*research@alsgbi.org*](mailto:research@alsgbi.org), +447973232038

**Supplementary Figures:**

Supplementary Figure 1 Google forms ALSGBI survey on training accessibility template.

Supplementary Figure 2 Survey monkey ALSGBI survey on robotic platform provision template (see separate PDF upload)

Supplementary Figure 3 Deanery by percentage response.

Supplementary Figure 4 Perception of a laparoscopic training curriculum by deanery.

Supplementary Figure 5 Access to laparoscopic simulation days by deanery.

Supplementary Figure 6 Access to laparoscopic training boxes by deanery.

Supplementary Figure 7 Access to robotic simulation by deanery.

Supplementary Figure 8 Access to robotic training days by deanery.

Supplementary Figure 9 Access to robotic lists by deanery.

Supplementary Figure 1 Google forms ALSGBI Survey template

Section 1 of 6

ALSGBI survey for minimally-invasive surgical training

Form description

After section 1

Continue to next section

Section 2 of 6

Survey Overview

Thank you for agreeing to help with this ALSGBI survey, we are grateful for your participation.

We hypothesise that access to, and frequency of, pre-clinical minimally-invasive training in Great Britain and Ireland is unequal across grade, specialty and location.

**Why complete this survey?**

We believe it is particularly pertinent given the training deficit created by COVID and anticipated with the rapid adoption of robotic systems.

**Purpose of this survey:**
To ascertain differences for trainees accessing minimally-invasive training facilities. Hopefully it's the first step to help secure funding and to aid standardisation of training.


Thanks, please don't hesitate to get in contact if you have any queries:

Matt Boal, ALSGBI trainee representative,

Vimaladhithan Mahendran, ALSGBI trainee representative,

Anuradha Thrikandiyur, ALSGBI trainee representative,

After section 2

Continue to next section

Section 3 of 6

ALSGBI Survey

Description (optional)

Which deanery are you reporting for?

*

Please type your age

*

Short-answer text

Gender

*

Other…

Which specialty are you reporting for?

*

Other…

What is your current level?

*

Other…

After section 3

Continue to next section

Section 4 of 6

Deanery specific

All questions in this section relate to Deanery wide training

Is there a laparoscopic training curriculum within your deanery?

*

Do you have regular laparoscopic training days in the clinical year?

*

If so, how many per year?

*

Laparoscopic training type

*

Do the deanery provide access to laparoscopic training boxes at home?

*

If so, what type?

*

If so, for how long?

*

Are there robotic training days within the year?

*

Do you have access to robotic VR simulation?

*

If so, how often per year?

*

After section 4

Continue to next section

Section 5 of 6

Hospital specific

This section relates to your current hospital, please state the name AND trust

Hospital Name and Trust

*

Short-answer text

Does the hospital provide regular laparoscopic training days in the clinical year?

*

If so, how many a year?

*

If so, what type of training?

*

Do you have access to lap training boxes within the hospital?

*

If so, what type?

*

If so, is there out of hours access?

*

Is there a surgical robot in this hospital?

*

If so, what type?

*

Other…

Is there robotic simulation available for training?

*

If so, is there access for trainees?

*

Do trainees have access to robotic lists?

*

If so, how often?

*

After section 5

Continue to next section

Section 6 of 6

Any additional information?

Thank you for filling out this survey. Please provide any additional information relating to laparoscopic and robotic training in your area

Is there any additional relevant information not covered by earlier sections?

*

Long-answer text

Supplementary Figure 3 Deanery by percentage response.

Supplementary Figure 4 Perception of a laparoscopic training curriculum by deanery

Supplementary Figure 5 Access to laparoscopic simulation days by deanery

Supplementary Figure 6 Access to laparoscopic training boxes by deanery

Supplementary Figure 7 Access to robotic simulation by deanery

Supplementary Figure 8 Access to robotic training days by deanery

Supplementary Figure 9 Access to robotic lists by deanery

**Supplementary Tables**

Supplementary Table 1: All specialties access to laparoscopic training curriculum

Supplementary Table 2: All specialties access to laparoscopic simulation days

Supplementary Table 3: All specialties access to laparoscopic training boxes

Supplementary Table 4: All specialties access to robotic simulation

Supplementary Table 5: All specialties access to robotic training days

Supplementary Table 6: All specialties access to robotic operating lists

Supplementary Table 1: All specialties access to laparoscopic training curriculum

| Specialties | Access to Lap Training Curriculum | | |
| --- | --- | --- | --- |
|  | YES | NO | DON'T KNOW |
| General Surgery | 43 | 53 | 48 |
| Urology | 3 | 4 | 2 |
| Gynaecology | 1 | 1 | 2 |
| Paediatric Surgery | 1 | 3 | 0 |
| ENT | 1 | 0 | 0 |
| Cardiothoracics | 0 | 1 | 0 |
| Other | 1 | 1 | 5 |

Supplementary Table 2: All specialties access to laparoscopic simulation days

| Specialties | Access to Lap Simulation Days | | |
| --- | --- | --- | --- |
|  | YES | NO | DON’T KNOW |
| General Surgery | 61 | 73 | 10 |
| Urology | 4 | 4 | 1 |
| Gynaecology | 2 | 2 | 0 |
| Paediatric Surgery | 2 | 2 | 0 |
| ENT | 1 | 1 | 0 |
| Cardiothoracics | 0 | 1 | 0 |
| Other | 1 | 3 | 2 |

Supplementary Table 3: All specialties access to laparoscopic training boxes

| Specialties | Access to Lap Training Boxes | | |
| --- | --- | --- | --- |
|  | YES | NO | DON’T KNOW |
| General Surgery | 83 | 36 | 20 |
| Urology | 5 | 3 | 1 |
| Gynaecology | 3 | 1 | 0 |
| Paediatric Surgery | 0 | 4 | 1 |
| ENT | 0 | 0 | 0 |
| Cardiothoracics | 0 | 0 | 0 |
| Other | 0 | 4 | 3 |

Supplementary Table 4: All specialties access to robotic simulation

| Specialties | Access to Robotic Simulation | | |
| --- | --- | --- | --- |
|  | YES | NO | DON'T KNOW |
| General Surgery | 18 | 47 | 15 |
| Urology | 3 | 3 | 3 |
| Gynaecology | 0 | 2 | 1 |
| Paediatric Surgery | 0 | 4 | 0 |
| ENT | 1 | 0 | 1 |
| Cardiothoracics | 0 | 0 | 0 |
| Other | 2 | 1 | 3 |

Supplementary Table 5: All specialties access to robotic training days

| Specialties | Access to Robotic Training Days | | |
| --- | --- | --- | --- |
|  | YES | NO | DON'T KNOW |
| General Surgery | 21 | 107 | 17 |
| Urology | 5 | 3 | 1 |
| Gynaecology | 1 | 2 | 1 |
| Paediatric Surgery | 0 | 4 | 0 |
| ENT | 1 | 1 | 0 |
| Cardiothoracics | 0 | 1 | 0 |
| Other | 0 | 2 | 3 |

Supplementary Table 6: All specialties access to robotic operating lists

| Specialties | Access to Robotic Lists | | |
| --- | --- | --- | --- |
|  | YES | NO | DON'T KNOW |
| General Surgery | 53 | 44 | 44 |
| Urology | 8 | 0 | 1 |
| Gynaecology | 1 | 3 | 0 |
| Paediatric Surgery | 1 | 2 | 1 |
| ENT | 2 | 0 | 0 |
| Cardiothoracics | 0 | 0 | 1 |
| Other | 1 | 1 | 4 |
